# Competency-Based Workforce Development and Education in Global Oncology

**DOI:** 10.3390/curroncol30020136

**Published:** 2023-02-01

**Authors:** Nazik Hammad, Ntokozo Ndlovu, Laura Mae Carson, Doreen Ramogola-Masire, Indranil Mallick, Scott Berry, E. Oluwabunmi Olapade-Olaopa

**Affiliations:** 1 Department of Oncology, Queen’s University, Kingston, ON K7L 3N6, Canada; 2 Department of Oncology, Faculty of Medicine and Health Sciences, University of Zimbabwe, Harare P.O. Box MP 167, Zimbabwe; 3 Division of Radiotherapy and Oncology, Parirenyatwa Group of Hospitals, Harare P.O. Box CY 198, Zimbabwe; 4 Department of ObGYN, Faculty of Medicine, University of Botswana, Private Bag UB 0022, Gaborone, Botswana; 5 Department of Radiation Oncology, Tata Medical Center, Kolkata 700 160, West Bengal, India; 6 Department of Surgery, College of Medicine, University of Ibadan, Ibadan P.M.B 5017, Nigeria

**Keywords:** medical education, competency-based medical education, oncology, global oncology, oncology workforce development

## Abstract

The healthcare workforce plays a pivotal role in cancer care delivery, leadership, policy, education, and research in complex cancer systems. To ensure quality and relevance, health professionals must have the necessary competencies to deliver patient-centered and efficient care, coupled with the ability to work in teams and manage health resources wisely. This paper aims to review the concept of competency-based medical education (CBME) in the context of oncology to provide insights and guidance for those interested in adopting or adapting competency-based education in training programs. The results of a scoping review of CBME in oncology are presented here to describe the current status of CBME in oncology. The literature describing the implementation and evaluation of CBME in oncology training programs for medical professionals internationally is summarized and key themes identified to provide practical guidance for educators. Further, the paper identifies critical competencies for oncology education and training globally and presents recommendations and opportunities for collaboration in competency-based education and training in oncology. The authors argue for increased global collaboration and networking in the realm of CBME to facilitate the establishment of a competent global cancer care workforce.

## 1. Introduction

The healthcare workforce plays a pivotal role in cancer care delivery, leadership, policy, education, and research in complex cancer systems. The challenge of inadequate human resources remains one of the most important obstacles to closing the equity gap in cancer care globally. According to the World Health Organization (WHO), there are three dimensions to the definition of transformative upscaling of health professional education: quantity, quality, and relevance [1]. To ensure quality and relevance, health professionals must have the necessary competencies to deliver patient-centered and efficient care, coupled with the ability to work in teams and manage health resources wisely [1]. Given that cancer is one of the diseases with the highest burdens of suffering and costs for patients, their families and communities, and the health care system, optimizing the workforce remains an urgent requirement [2]. Delivery of high-quality, value-based cancer care requires technical expertise and knowledge, including critical appraisal of evidence, in addition to competencies in communication, advocacy, leadership, professionalism, life-long learning, and scholarship. Implementing competency-based medical education is one strategy that has the potential to foster the development of these critical skills among cancer care providers.

This paper aims to review the concept of competency-based medical education in the context of oncology, as well as provide insights and guidance for those interested in adopting or adapting competency-based education in training programs. First, we will review the concept and describe the importance of competency-based medical education and training in oncology. Then, we will describe the results of a scoping review of CBME in oncology to understand the state of the existing literature on competency-based frameworks in oncology health care profession training before focusing on medical professional training specifically. The literature describing the implementation and evaluation of CBME in oncology training programs for medical professionals will be summarized and the key themes emerging, gaps in literature and areas of uncertainties will be described to help guide oncology educators and identify gaps that need to be addressed. Next, we will identify the critical competencies for oncology education and training. Finally, we will outline future directions and opportunities for collaboration in competency-based education and training in oncology.

### Current Status of Competency-Based Medical Education

The goal of medical education is to produce graduates with the knowledge and understanding of clinical and non-clinical sciences who have acquired the essential skills and attitudes required for high standards of medical practice in modern health systems [3]. In light of this, there is a global shift to train physicians with easily identifiable and measurable competencies who effectively meet the needs of the populations they serve [4]. Therefore, outcome-based curricula focusing on education models that can respond to workforce needs and the rapidly changing health needs of individual communities [5] are increasingly being adopted worldwide.

Competency-based medical education (CBME) is a collection of pedagogical principles and approaches that are constantly evolving to meet the primary aim of achieving better outcomes for learners and teachers with the ultimate goal of improving patient outcomes [6]. It is an outcomes-based approach to the design, implementation, and evaluation of education programs and teaching and assessment methods across the medical continuum that uses competencies or observable abilities of learners [7]. In contrast to time-based educational methods, CBME is a learner-centered, active, and lifelong learning experience that incorporates feedback between the teacher and the learners to attain the desired competency outcomes. It de-emphasizes time-based training and promises greater accountability, flexibility, and learner-centeredness [8]. “The intended outcome [of CBME] is a health-professional who can practice medicine at a (globally) defined level of proficiency, in accord with local conditions, to meet local needs” [9]. In addition to introducing CBME in undergraduate medical instruction, it is being introduced to postgraduate medical education (PGME) programs worldwide to facilitate medical specialties training and assessment that result in the most meaningful competencies for the discipline and local context [10,11,12]. In a recent follow-up review to the seminal 2010 Lancet Commission [13], Frenk et al. note that, “Competencies for professional work have increasingly become accepted as the optimal outcome of health- professional education [13]. See Appendix A for a framework for developing a CBME curriculum.

Competency-based education models, however, have limitations, as they may be too narrowly focused on individual abilities [14] and may be misapplied by inadequately and or inappropriately trained teachers [15,16]. Thus, sensitization of stakeholders and faculty development is critical to improve the acceptance and ensure effective implementation across medical schools [17]. Trainers and trainees must have a good understanding of the rationale and methods of outcome-based instruction to ensure graduates have benefited fully from this framework. See Box 1 for a list of these components, and the five essential elements of CBME.

Box 1 **Components of CBME and The Five Essential Elements of CBME**

Components of CBME

1. Competencies—These are the essential attitudes, skills and knowledge required to carry out specific tasks in sequential grades of expertise [13]. However, medical practice requires the physician to independently integrate competencies from several competency domains in multiple combinations to suit the needs of individual patients [18]
2. Entrustable professional activities (EPAs)—These are globally accepted specific professional tasks the public expects all physicians to be able to carry out independently upon graduating and consist of real-life physician tasks and which have measurable outcomes [14]. EPAs are increasingly being defined for training in individual health professional specialties [15] and have recently been defined for newly graduated physicians [19]
3. Essential Professional Duties—These are groups of EPAs directed at carrying out a particular recognized professional duty effectively in a specified location [20,21], i.e., locally relevant professional activities of international standard that represent identifiable outcomes against which the effectiveness of professionals in a specific community can be measured to ensure social responsiveness and accountability.

The Five Essential Elements of CBME

The five essential and indispensable/core elements of CBME include [22]

(1) Clearly articulated outcome competencies required for practice
(2) Sequenced progression of competencies and their developmental markers
(3) Tailored learning experiences that facilitate the acquisition of competencies
(4) Competency-focused instruction that promotes the acquisition of competencies
(5) Programmatic assessment

## 2. Scoping Review: Competency-Based Medical Education (CBME) in Oncology

A scoping review was conducted to evaluate the literature on competency-based medical education in oncology. The search was performed using PubMed for the years 1992 to 2022. Position articles, commentaries, and editorials were excluded. The following types of articles were included: articles that outlined the development of oncology-related competencies; articles that provided evaluations of competency-based medical education or training programs and/or their implementation; articles that presented a list of oncology-related competencies; articles that reported on needs assessments related to competency-based training or education in oncology. The initial search returned 737 results. After the initial exclusion of duplicates, titles and abstracts of 735 articles were reviewed for inclusion. Two hundred and four articles were identified for full-text review. The total number of articles included after the full-text review was 117. Refer to Appendix B and Appendix C for the search strategy and PRISMA diagram. Given the paucity of literature on CBME in low- and middle-income countries (LMICs), we relied on a broad search strategy coupled with the use of gray literature in other sections of the paper to glean the most comprehensive data on the subject.

Sixty papers (51%) were related to medical education, that is the focus of this paper followed by nursing (37%) (Table 1). Radiation oncology (27%) was the medical discipline with the most articles identified. Over a dozen articles (10%) referenced the CANMEDS framework as a guide to their curricular development, an educational framework including principles of CBME designed by the Royal College of Physicians and Surgeons of Canada [23]. Less than 20% of the included articles had a focus or included LMICs.

The countries that had representation in the highest number of included articles were the United States (47), Canada (40), the UK (15), Australia (12), and Italy (7). Belgium, Denmark, and Germany had seven articles each, and France, the Netherlands, Switzerland each had five. Brazil, New Zealand and Spain had four articles each, whereas Austria, Greece, Norway, Portugal, and Sweden had three. Countries with two articles were China, Croatia, Egypt, India, Israel, Malaysia, Mexico, Poland, Rwanda, Singapore, and South Africa. Finally, countries that were represented by one article were Albania, Botswana, Chile, Czech Republic, Estonia, Finland, Ghana, Guatemala, Iran, Ireland, Japan, Jordan, Latvia, Malawi, Nigeria, Romania, Senegal, Slovenia, South Korea, Taiwan, Tanzania, Vietnam, Zambia, and Zimbabwe.

**Figure 1:**
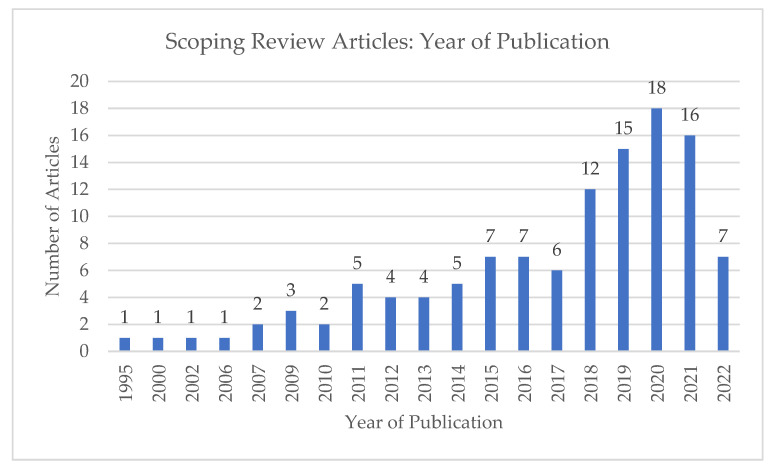
Year of publication of scoping review articles.

The included articles were separated into three categories: Needs assessments, program/implementation evaluations, or competency outlines (which either presented a list of competencies or reviewed how such a list was developed). Most articles (54.9%) were competencies outlines. Program evaluation and needs assessment articles constituted 23.9% and 21.2% of included articles, respectively. The results of the scoping review are summarized in Table 1 and Figure 1.

## 3. Implementation and Evaluation

CBME has many theoretical benefits, but these will never be realized if programs are not adequately designed, implemented, or evaluated. The scoping review identified eight studies related to the design, implementation, or evaluation of CBME. The studies and key findings are in Table 2.

Turner et al.’s study of CBME design, implementation, and evaluation was the most extensive study identified in our review and presented the most mature data [29]. The authors reported that over 90% of participants claimed that their implementation of CBME in radiation oncology programs across Australia and New Zealand “provided direction in attaining competencies”. Two-thirds of respondents, including trainees, felt that “work readiness” was improved. Furthermore, 81% of respondents indicated that their implementation of CBME “promotes regular, productive interaction between trainees and supervisors” and the majority found the quality of feedback above average or excellent.

Some important common themes emerged from this and other smaller studies:

### 3.1. Importance of Faculty and Learner Development

Several studies highlighted the importance of learner development [24], faculty development [25], or both [26]. The concept of leadership as a form of development was also referenced.

### 3.2. Logistical and Other Obstacles

Several logistical and technical obstacles were identified by Arora [24] (time), Tomiak [25] (time, integrating into clinical workflow); Tomiak [26] (time, assessment platform); Moideen [27] (time); and Safavi [28] (time, integrating into clinical workflow, assessment tools). Notwithstanding these issues, Turner and colleagues reported that nearly two-thirds of respondents in their study stated that clinical service requirements could be met during training.

### 3.3. Importance of Program Structure

Structure was also frequently discussed as a logistical element of competency-based programs. Many evaluations emphasized that having a defined program structure with formal milestones, EPAs, and/or a clearly defined set of structured competencies was essential for program success [32,33].

### 3.4. Opportunities for Change

Arora et al. reported that all program directors involved in implementing CBME in medical oncology programs in Canada used this as an opportunity to revise the structure and sequence of clinical rotations [24]. Arora [24] also reported on the introduction of new training experiences and techniques with CBME implementation, including 80% who were introducing new electronic teaching modules to supplement resident learning. Participants in Moideen et al.’s study suggested the development of case libraries and computer-based clinical vignettes as innovations that could aid the implementation of CBME for radiation treatment planning [27].

An article by Canadian medical oncology educational leaders involved in the design and implementation of CBME for Canadian medical oncology trainees provided 10 pieces of guidance based on a review of the available literature and the authors’ experiences to help educators implement CBME. They stressed the importance of involving key stakeholders, such as trainees, teaching faculty, residency training committee members, and the program administrator, prior to and throughout the implementation of CBME that was highlighted in the studies above. The authors believed that the careful and selective choice of crucial design elements, including EPAs, assessments, and appropriate use of direct observation would enhance the successful uptake of CBME. They suggested that pilot testing may help engage faculty and learners and identify logistical issues that may hinder implementation [34].

There is a paucity of data regarding CBME in oncology in LMICs. Kiguli-Malwadde et al. summarize the process of revising a traditional discipline-based curriculum and developing and implementing a CBME curriculum at two sub-Saharan medical schools for their undergraduate training programs [5]. Lessons from this study—including the importance of considering local context in the development of CBME curricula and involving a diverse group of stakeholders—can be used in developing and implementing CBME in oncology in LMICs. In their paper from Zimbabwe, Africa, Ndlovu et al. described the process of transitioning their clinical oncology program from a traditional-based training program to a CBME one based on the findings from Turner et al. [30,35]. Chinula and colleagues in Malawi showed how CBME principles could be customized to meet the unique challenges in LMICs where formal oncology training programs are limited by creating shorter and specific skill-based training to respond to high-burden diseases such as cervical cancer [31].

Most of the studies evaluating implementation and evaluation of CBME identified were limited by their small size. There was also limited geographic representation, especially of LMICs, with only 22% based in LMICs. The insights are helpful but need to be interpreted and applied based on local context. Oncology educators who have implemented or are in the process of implementing CBME should be encouraged to critically evaluate and publish their experiences to help guide other educators and identify the most successful strategies and common pitfalls to ensure that CBME can realize its potential. Despite these limitations, the themes that emerged from the oncology-focused literature on the implementation of CBME were similar to those identified in a recent narrative review on CBME implementation [36].

### 3.5. Competencies in Global Oncology

Several academic bodies and professional cancer societies have implemented competency-based education (Appendix D). Further, many of these have embarked on developing a set of competencies in “global oncology” for their learners. These competencies are mostly designed to guide and increase the global relevance of high-income country (HIC)-based researchers’ and practitioners’ engagement with cancer care and research in LMIC. For example, the global curriculum in Medical Oncology published by the European Society of Medical Oncology (ESMO) together with the American Society for Clinical Oncology (ASCO) published in 2016 [37] contains sections outlining the unique needs of oncology practice in LMICs. This section includes an understanding of the differences in the etiology of cancers in low-resource settings, approaches to prevention, available treatment options, and outcomes of treatment. It also includes knowledge of the WHO essential medicines list, and resource stratified guidelines in cancer care where available.

## 4. Critical Competencies Addressing Challenges of Cancer Care in Both HIC and LMIC

Several critical oncology competencies and competency areas have relevance for all regions and cancer care providers. Upon reviewing the literature, including published needs assessments, expert agency guidelines, and educational documents, critical competencies that need to be better integrated into oncology training were selected for discussion here. These competencies are widely understood as integral to high-quality cancer care delivery and must be considered in developing oncology curricula for education or workforce development programs.

### 4.1. Value-Based Care

Given the high societal and individual costs of new therapies, often for limited benefit, one of the key elements of oncology in both HICs and LMICs is identifying the cost-benefit of treatments in the context of the society being served. According to the WHO 2020 Report on Cancer, “setting priorities, investing wisely and providing care for all” “medical oncology services must be strengthened in steps, according to the health system requirements and the impact and cost of treatments, some of which may offer little benefit to patients and have high social costs” [38]. Learners should be competent in evaluating the value of treatments with tools including or analogous to the ASCO value framework [39] and/or the ESMO Magnitude of Clinical Benefit Scale [40]. There is also a need to avoid low-value diagnostic and treatment practices. The Choosing Wisely recommendations in different geographic regions in LMICs provide important recommendations in this respect [41,42,43,44].

### 4.2. Integrative Oncology

Integrative oncology has become more pertinent as more patients use complementary and alternative therapies [45]. Training on these therapies and interventions is critical in countering the heterodoxy of the “Western” medical model and associated colonial trappings that may discount local knowledge and practices that may enhance quality of life or increase the uptake of interventions across the continuum. These alternative interventions come in many forms depending on the setting and can range from lifestyle modifications to physical activity to mind-body and spiritual interventions. The aim would be to train oncologists who recommend evidence-based integrative oncology therapies alongside conventional cancer treatments to their patients. Innovative pedagogy may be needed to teach such competencies.

### 4.3. Technology-Enhanced Education

The adoption of technology-enhanced education methodology is increasingly becoming the future route in radiation oncology, largely due to the phenomenal development of artificial intelligence in the field. This can be employed to address the additional clinical competencies that may be required [46,47]. Online learning helps to address faculty shortage in delivering some of the required training experiences in CBME. With the increasing uptake of technology in LMIC, more research is needed on the feasibility and the harnessing of technology for CBME implementation.

### 4.4. Leadership

In their recent review, Frenk et al. highlighted that a critical competency for all heath care professionals is leadership ^13^. In oncology, leadership is an essential competency that needs particular focus for development. In our scoping review, seven included articles had an explicit focus on the concept of leadership as a competency, and several others mentioned leadership in their lists of competencies [48,49]. Medical specialists have an important role in leadership within the healthcare systems, making competency-based curricula for leadership in post-graduate specialty training essential. Clinicians have been shown to be influential in health system decisions, such as healthcare expenditure and policy-making [50]. As such, clinicians assume a leadership role that directly influences their health system. Offering quality leadership training can improve patient outcomes and the professional environment and strengthen and advance entire health systems. Ideally, leadership training should be discipline-specific, but curricula that include this are limited and need to be better developed across all areas of medical specialty training [51]. Many oncology training programs in LMIC place greater emphasis on leadership, as workforce shortages demand these competencies [30]. Many learners in these programs are likely to work as independent practitioners who will need to act as experts, leaders, collaborators, and advocates for developing cancer care systems within their spheres of influence [52]. The goal of CBME is to train oncologists who have the necessary skills and abilities to transition into independent practice seamlessly. Notably, a global survey of practicing oncologists and trainees in both HIC and LMIC revealed that oncologists felt the least prepared for practice in competencies related to leadership, effective management of oncology practice, and understanding of healthcare systems [53]. Several gaps have been reported in this transition-to-practice in radiation oncology, including a lack of experience with practice management, financial planning and limited understanding of the structure and function of the healthcare system [54]. Ensuring training programs include training in competencies that enable oncologists to effectively lead their own practices while effectively and constructively influencing the health systems they work in is essential.

### 4.5. Health Equity

In their recent review, Frenk et al. comment that in competency-based education, “health equity is increasingly recognised as a neglected curricular theme amid substantial health disparities among population subgroups, defined by urban or rural residence, race, ethnicity, income, housing, and education” [13]. Including competencies and meaningful instruction in recognizing disparities and improving health equity is important for all health care professionals including oncologists.

## 5. Relevance for LMICs

By 2040, 67% of annual cancer cases will be in LMIC [38]. The shortage of oncologists and other cancer health professionals remains a major obstacle to delivering high-quality care. In LMICs, CBME in oncology could be more impactful for several reasons. Oncology practitioners often work as single practitioners in one or a few centers in a country. They often perform as clinical oncologists trained in administering radiotherapy and chemotherapy treatment. They are also expected to be involved in cancer control activities and are the leads in cancer service provision [52]. Resource-limited regions often have weak infrastructure, limiting the potential to integrate clinical and technological advances, which can support clinical competency development. CBME with its emphasis on life-long learning and learner centeredness can help enhance self-directed learning and knowledge sharing in such settings. Stewardship of limited resources requires several competencies in leadership, advocacy, and communication in addition to value-based care.

There are significant areas of uncertainties that should become a focus of critical inquiry, such as the type of assessments and costs of CBME in both HIC and LMIC and whether it is feasible to implement the full array of CBME components such as EPAs and milestones in LMICs. Current available literature does not provide evidence of implementation of EPAs in LMIC oncology training programs; however, this does not exclude that they are currently being or will be implemented in the future. In addition, the shortage of cancer health professionals has created an imperative for task shifting in cancer care, especially in LMIC, involving scaling-up the capacity for core competence, such as in palliative care [38]. However, caveats include the availability of supportive supervision by the task shifters to whom the task is shifted. As the WHO points out, there is little or no evidence on whether non-specialists can safely and effectively prescribe systemic therapy or radiotherapy [38]. Continuing faculty development is essential to improve perceptions and identify best practices and expectations for the discipline may be further required [28].

Despite resource constraints and other challenges in CBME implementation in oncology training programs in LMICs, some successful innovative programs have been reported. For example, Ndlovu and colleagues successfully transitioned a clinical oncology program to a CBME-based program in Zimbabwe, and Khader et al. reported on the successful development of a competency-based residency training program in radiation oncology in Jordan [30,55]. Many training programs have syllabi that lack key CBME components such as competency mapping, EPAs, or milestones. Trainers in institutions planning to implement CBME should keep this in mind and prepare for curricular modifications in the process. As noted above, there is currently a paucity of published studies on the implementation, evaluation, and tracking of outcomes of CBME in oncology based in LMICs. Research and description of CBME adoption in LMIC is of paramount importance while its implementation is still evolving.

## 6. Opportunities for Collaboration and Way Forward

Cancer care and its societal costs present a major global challenge in LMIC and HIC. There are enormous opportunities for shared global learning endeavors in implementing and evaluating CBME in both HIC and LMIC. This will require South–South and North–South collaboration and equitable partnerships. While the WHO has called for the adoption of CBME since the 1970s [1,9], global collaborations in CBME have been limited and are mostly tied to accreditation pursuits in some higher-income countries in the global south, such as the Gulf states or Singapore [56]. The roster for the International Competency-based Medical Education (ICBME) collaborators who examine conceptual issues and current debates in CBME does not have a single member from LMIC [57]. Given that the CBME mandate is global (WHO-based) and has been embraced by a significant number of LMICs and that the global cancer community is increasingly interconnected, cancer health professional education has a unique opportunity to decolonize the CBME debate and creating a shared learning global movement in which knowledge produced in LMICs can inform best practices in both HIC and LMIC settings. This collaboration will allow for the sharing of resources in implementation, curriculum design, and assessment and ultimately may shed light on whether CBME will deliver on its ultimate promise of improving health outcomes at the level of the individual patient and general population.

CBME as an educational innovation, has seen significant cross-cultural adaptation and contextualization. For example, CanMEDS—a framework that employs principles of CBME—has been adopted by many countries in sub-Saharan Africa in specialties such as the eye workforce and other medical specialties [58,59].

The centrality of the workforce in delivering equitable cancer care and accelerating progress towards Universal Health Coverage (UHC) and the Sustainable Development Goals (SDGs) has been increasingly recognized [38,60]. However, research on health professional education is lagging significantly behind other areas of cancer research in terms of funding and recognition. Strengthening research on health professional education and workforce optimization is often omitted, even in the most recent calls delineating the priorities for cancer research in LMIC [61]. Global collaboration in CBME can hopefully raise the profile of educational scholarship, address areas of uncertainty, and stimulate the CBME debate and the global sharing of best practices in optimizing a cancer workforce capable of delivering compassionate and competent care while ensuring good stewardship of resources across the globe. Vehicles for such collaboration include enhancing partnerships between academic institutions, utilizing the platforms of oncology professional organizations and societies, and creating global oncology CBME networks and bodies.

## Figures and Tables

**Table 1:** Scoping Review Summary.

Category	Number of Included Articles (%)
Competency Outlines	64 (54.7)
Program Evaluation	28 (23.9)
Needs Assessment	25 (21.4)
Professional Category *Note: Oncology nursing was counted as “nursing”	Number of Included Articles (%)
Oncology-Related Medical Disciplines	63 (53.8)
*Clinical Oncology*	*3 (2.6)*
*General Oncology*	*12 (10.3)*
*Global Oncology*	*1 (0.9)*
*Gynecologic Oncology*	*2 (1.7)*
*Medical Oncology*	*4 (3.4)*
*Neuro-Oncology*	*1 (0.9)*
*Pediatric Oncology*	*4 (3.4)*
*Psychosocial Oncology*	*1 (0.9)*
*Radiation Oncology*	*27 (23.1)*
*Surgical Oncology*	*5 (4.3)*
*Hematology*	*3 (2.6)*
Nursing	37 (31.6)
Medical Physics	6 (5.1)
General Medicine	4 (3.4)
Community/Public Health	2 (1.7)
Palliative Care	2 (1.7)
Research	2 (1.7)
Genomics	1 (0.9)
Massage Therapy	1 (0.9)
Multiple	1 (0.9)
Patient Educators	1 (0.9)
Pharmacy	1 (0.9)

**Table 2:** Scoping review: papers describing implementation and evaluation of CBME for oncology medical professional training programs.

Specialty	Author(s)	Country	Study Type	Size	Key Findings
Medical Oncology	Arora 2020 et al. [24]	Canada	National Pre-CBME Implementation Survey	14/15 Program Directors	Describes major structural and curricular changes while transitioning to CBME including Modifications to clinical (a) rotations (b) Heme (c) RO (d) progression of competence Changes to enhance resident learning (a) electronic teaching modules (b) new training experiences (c) changes to didactic teaching sessions)
	Tomiak 2020 et al. [25]	Canada	Single Institution Mixed Methods Pre CBME Implementation Pilot	17 workplace-based assessments Resident Focus Group (*n* = 4) Faculty Interviews (*n* = 5)	Identified 9 lessons learned during implementation in Canadian Med Onc Program (1) faculty and resident development and engagement; (2) sharing the work of CBME; (3) collaboration and communication; (4) global assessment; (5) assessment plan challenges; (6) burden of CBME; (7) limitations of e- portfolio; (8) importance of early tracking of resident progress; and (9) culture change
	Tomiak 2020 et al. [26]	Canada	Single Institution Retrospective Review of First Year of CBME Implementation	157 Assessments by 9 Faculty	Six main findings: (1) Verbal feedback is preferred over written; (2) Providing both written and verbal feedback is important; (3) Effective feedback was seen as timely, specific, and actionable; (4) The process was conceptualized as coaching rather than high stakes; (5) There were logistical concerns about the WBAs, and additional clarification about the WBA tools is needed.
Radiation Oncology	Moideen 2019 et al. [27]	Canada	Single Institution Qualitative Study	11 Radiation Oncologists 7 Residents 7 Dosimetrists	3 Themes: (1) Strengths of treatment planning in CBME: Challenges of treatment planning in CBME, Competency-based assessments enrich student learning, Increased engagement in the feedback process will act as a catalyst for more useful and frequent feedback. (2) Challenges of treatment planning in CBME Workload demands, Clear expectations for competency at each training stage, Need for systemic cultural change (3) Opportunities for change Development of a library of cases, Structured formative treatment planning assessments, Innovative teaching and learning strategies to support the development of quality treatment plans
	Safavi 2021 et al. [28]	Canada	Single Institution Mixed Methods Implementation Pilot	7 Radiation Oncologists 6 Residents	Three Themes: (1) Direct observation is the most challenging aspect of CBD to implement; (2) feedback content can be improved; and (3) staff attitude, clinical workflow, and inaccessibility of assessment forms are the primary barriers to completing assessments
	Turner 2015 et al. [29]	Australia New Zealand	National External Independent Mixed Methods Evaluation	35/45 Training Sites 200 Faculty Interviews 119 Faculty Survey Respondents 80 Faculty Interviews 38 Faculty Survey Respondents	Over 90% responding that it ‘provided direction in attaining competencies’. Most (87/107; 81%) said it ‘promotes regular, productive interaction between trainees and supervisors’. Adequacy of feedback to trainees was rated as only ‘average’ by trainees/trainers in one-third of cases. Consultations revealed this was more common where trainers were less familiar with curriculum tools. Half of training directors/supervisors felt better supported. Nearly two-third of all responders (58/92; 63%) stated that clinical service requirements *could* be met during training; 17/92 (18.5%) felt otherwise. When asked about ‘work-readiness’, 59/90 (66%) respondents, including trainees, felt this was improved.
Clinical Oncology	Ndlovu 2021 et al. [30]	Zimbabwe	National Description of the CO programme and its progression from knowledge-based to competency-based	Not applicable	The curriculum is being reviewed working towards standardizing it across the African context and including domains of competency skills such as: clinical decision-making, communication, knowledge, attitude required for the above appropriately.
Specific surgical skills training in cervical cancer treatment	Chinula 2018 et al. [31]	Malawi	National A description of a narrow, specific competency-based skills training for performing radical abdominal hysterectomy and bilateral pelvic lymphadenectomy in a relatively short period of time	Performed at Kamuzu Central Hosp, a 1000-bed teaching hospital in Lilongwe. Board certified Malawian ObGyns trained by two US-board certified gyn-oncology master trainers.	Self-directed learning; Onsite training; Intraop assessment of tech skills; Continued E-learning with master trainer. During first 24 months of programme 28 patients underwent surgery by one trainee. During the first 5-day practicum 7 cases operated on by trainee and master trainer; 8th case performed exclusively by unsupervised trainee on the last day; 20 cases operated on independently by trainee over the course of 24 months.

## **Appendix B. Scoping Review Strategy**

PUBMED was searched on 4/27/2022

Search Terms: ((“competency-based”) OR (“competencies”)) AND (oncology)

## **Appendix D. Table of Selected Available CBME Curricula in Medical Oncology and Clinical Oncology**

**Associations**	**Target Audience**	**Competency Framework**	**Integrated into Curriculum?**	**Implementation and Assessment**
Joint Royal College of Physicians Training Board [62]	Medical Oncology the UK 2017	General Medical Council (GMC) Good Medical Practice (GMP) and Medical Leadership Competency Framework	Yes	UK
Clinical Oncology [63]	Royal College of Radiologists	Generic Professional capabilities. Rather than competencies: Capabilities in practice (CiP) GPC	Yes	UK
Medical Oncology Ireland: Royal College of Physicians of Ireland (RCPI): Irish Committee on Higher Medical Training version 2018 [64]	Medical oncology	Good Medical Practice (GMP)	Yes	Ireland
ASCO/ACGME medical oncology training [65]	Medical oncology 2021	ACGME competencies	Yes	USA and programs in other countries accredited by ACGME International
The Royal Australian College of Physicians Australia [66]	Medical Oncology 2013	The Professional Qualities Curriculum (PQC) In addition to Specific medical oncology domains and expected outcomes	Yes	Australia

## References

[1] World Health Organization (2013). Transforming and Scaling up Health Professionals’ Education and Training: World Health Organization Guidelines 2013.

[2] Aggarwal A., Lievens Y., Sullivan R., Nolte E. (2022). What Really Matters for Cancer Care-Health Systems Strengthening or Technological Innovation?. Clin. Oncol..

[3] Kwizera E., Iputo J. (2011). Addressing social responsibility in medical education: The African way. Med. Teach..

[4] Boelen C. (2004). Building a socially accountable health professions school: Towards unity for health. Educ. Health.

[5] Kiguli-Malwadde E., Omaswa F., Olapade-Olaopa E.O., Kiguli S., Chen C., Sewankambo N.K., Ogunniyi A.O., Mukwaya S. (2014). Competency-based medical education in two Sub-Saharan African medical schools. Adv. Med. Educ. Pract..

[6] Holmboe E.S., Sherbino J., Englander R., Snell L., Frank J.R., Collaborators O.B.O.T.I. (2017). A call to action: The controversy of and rationale for competency-based medical education. Med. Teach..

[7] Association of American Medical Colleges (AAMC). https://www.aamc.org/what-we-do/mission-areas/medical-education/cbme.

[8] Frank J.R., Mungroo R., Ahmad Y., Wang M., De Rossi S., Horsley T. (2010). Toward a definition of competency-based education in medicine: A systematic review of published definitions. Med. Teach..

[9] McGaghie W.C., Sajid A.W., Miller G.E., Telder T.V., Lipson L., World Health Organization (1978). Competency-Based Curriculum Development in Medical Education: An Introduction.

[10] Englander R., Flynn T., Call S., Carraccio C., Cleary L., Fulton T.B., Garrity M.J., Lieberman S.A., Lindeman B., Lypson M.L. (2016). Toward Defining the Foundation of the MD Degree: Core Entrustable Professional Activities for Entering Residency. Acad. Med..

[11] Morrison K., MacNeily A. (2006). Core competencies in surgery: Evaluating the goals of urology residency training in Canada. Can. J. Surg..

[12] van Houwelingen C.T., Moerman A.H., Ettema R.G., Kort H.S., Ten Cate O. (2016). Competencies required for nursing telehealth activities: A Delphi-study. Nurse Educ. Today.

[13] Frenk J., Chen L.C., Chandran L., Groff E.O.H., King R., Meleis A., Fineberg H.V. (2022). Challenges and opportunities for educating health professionals after the COVID-19 pandemic. Lancet.

[14] Green M.L., Aagaard E.M., Caverzagie K.J., Chick D.A., Holmboe E., Kane G., Smith C.D., Iobst W. (2009). Charting the road to competence: Developmental milestones for internal medicine residency training. J. Grad. Med. Educ..

[15] Kogan J.R., Holmboe E., Hauer K. (2009). Tools for direct observation and assessment of clinical skills of medical trainees: A systematic review. JAMA.

[16] Hauer K.E., Kohlwes J., Cornett P., Hollander H., Cate O.T., Ranji S.R., Soni K., Iobst W., O’Sullivan P. (2013). Identifying entrustable professional activities in internal medicine training. J. Grad. Med. Educ..

[17] Olapade-Olaopa E.O., Fasola A.O., Agunloye A.M., Ogunbiyi A.O., Odukogbe A.A., Ogunniyi A.O. (2022). Revising a 60-Year Old Medical and Dental Curriculum in a Medical School in Sub-Saharan Africa [Manuscript accepted for publication January 25, 2022]. Afr. J. Med. Med. Sci..

[18] ten Cate O., Snell L., Carraccio C. (2010). Medical competence: The interplay between individual ability and the health care environment. Med. Teach..

[19] Sule H., Lamba S., Wilson B., Natal B., Anana M., Nagurka R. (2016). A suggested emergency medicine boot camp curriculum for medical students based on the mapping of Core Entrustable Professional Activities to Emergency Medicine Level 1 milestones. Adv. Med. Educ. Pract..

[20] Fujihara H., Koinuma M., Yumoto T., Maeda T., Kamite M., Kawahara E., Soeda S., Takimoto A., Tamura K., Nakamura M. (2015). Expected Duties of Pharmacists and Potential Needs of Physicians and Nurses on a Kaifukuki Rehabilitation Ward. YAKUGAKU ZASSHI.

[21] Olapade-Olaopa E.O., Sewankambo N., Iputo J.E., Rugarabamu P., Amlak A.H., Mipando M. (2016). Essential professional duties for the sub-Saharan medical/dental graduate: An Association of Medical Schools of Africa initiative. Afr. J. Med. Med. Sci..

[22] Van Melle E., Frank J.R., Holmboe E.S., Dagnone D., Stockley D., Sherbino J. (2019). A Core Components Framework for Evaluating Implementation of Competency-Based Medical Education Programs. Acad. Med..

[23] Royal College of Physicians and Surgeons (2022). CanMEDS Framework. https://www.royalcollege.ca/rcsite/canmeds/canmeds-framework-e.

[24] Arora R., Kazemi G., Hsu T., Levine O., Basi S.K., Henning J.W., Sussman J., Mukherjee S.D. (2020). Implementing changes to a residency program curriculum before competency-based medical education: A survey of Canadian medical oncology program directors. Curr. Oncol..

[25] Tomiak A., Braund H., Egan R., Dalgarno N., Emack J., Reid M.-A., Hammad N. (2020). Exploring How the New Entrustable Professional Activity Assessment Tools Affect the Quality of Feedback Given to Medical Oncology Residents. J. Cancer Educ..

[26] Tomiak A., Linford G., McDonald M., Willms J., Hammad N. (2020). Implementation of Competency-Based Medical Education in a Canadian Medical Oncology Training Program: A First Year Retrospective Review. J. Cancer Educ..

[27] Moideen N., de Metz C., Kalyvas M., Soleas E., Egan R., Dalgarno N. (2020). Aligning Requirements of Training and Assessment in Radiation Treatment Planning in the Era of Competency-Based Medical Education. Int. J. Radiat. Oncol. Biol. Phys..

[28] Safavi A.H., Sienna J., Strang B.K., Hann C. (2023). Competency-Based Medical Education in Canadian Radiation Oncology Residency Training: An Institutional Implementation Pilot Study. J. Cancer Educ..

[29] Turner S., Seel M., Berry M. (2015). Radiation Oncology Training Program Curriculum developments in Australia and New Zealand: Design, implementation and evaluation—What next?. J. Med. Imaging Radiat. Oncol..

[30] Ndlovu N., Ndarukwa S., Nyamhunga A., Musiwa-Mba P., Nyakabau A.M., Kadzatsa W., Mushonga M. (2021). Education and training of clinical oncologists-experience from a low-resource setting in Zimbabwe. Ecancermedicalscience.

[31] Chinula L., Hicks M., Chiudzu G., Tang J.H., Gopal S., Tomoka T., Kachingwe J., Pinder L., Hicks M., Sahasrabuddhe V. (2018). A tailored approach to building specialized surgical oncology capacity: Early experiences and outcomes in Malawi. Gynecol. Oncol. Rep..

[32] Petz W., Spinoglio G., Choi G.S., Parvaiz A., Santiago C., Marecik S., Giulianotti P.C., Bianchi P.P. (2016). Structured training and competence assessment in colorectal robotic surgery. Results of a consensus experts round table. Int. J. Med. Robot..

[33] Rodin D., Yap M.L., Grover S., Longo J.M., Balogun O., Turner S., Eriksen J.G., Coleman C.N., Giuliani M. (2017). Global Health in Radiation Oncology: The Emergence of a New Career Pathway. Semin. Radiat. Oncol..

[34] Hsu T., De Angelis F., Al-Asaaed S., Basi S.K., Tomiak A., Grenier D., Hammad N., Henning J.-W., Berry S., Song X. (2021). Ten ways to get a grip on designing and implementing a competency-based medical education training program. Can. Med. Educ. J..

[35] Turner S., Seel M., Trotter T., Giuliani M., Benstead K., Eriksen J.G., Poortmans P., Verfaillie C., Westerveld H., Cross S. (2017). Defining a Leader Role curriculum for radiation oncology: A global Delphi consensus study. Radiother. Oncol..

[36] Stoffman J.M. (2022). Overcoming the barriers to implementation of competence-based medical education in post-graduate medical education: A narrative literature review. Med. Educ. Online.

[37] Dittrich C., Kosty M., Jezdic S., Pyle D., Berardi R., Bergh J., El-Saghir N., Lotz J.-P., Österlund P., Pavlidis N. (2016). ESMO/ASCO Recommendations for a Global Curriculum in Medical Oncology Edition 2016. ESMO Open.

[38] World Health Organization (2020). WHO Report on Cancer.

[39] Schnipper L.E., Davidson N.E., Wollins D.S., Blayney D.W., Dicker A.P., Ganz P.A., Hoverman J.R., Langdon R., Lyman G.H., Meropol N.J. (2016). Updating the American Society of Clinical Oncology Value Framework: Revisions and Reflections in Response to Comments Received. J. Clin. Oncol..

[40] Cherny N.I., Dafni U., Bogaerts J., Latino N.J., Pentheroudakis G., Douillard J.-Y., Tabernero J., Zielinski C., Piccart M.J., de Vries E.G.E. (2017). ESMO-Magnitude of Clinical Benefit Scale version 1. 1. Ann. Oncol..

[41] Pramesh C.S., Chaturvedi H., Reddy V.A., Saikia T., Ghoshal S., Pandit M., Babu K.G., Ganpathy K.V., Savant D., Mitera G. (2020). “Choosing Wisely” for Cancer Care in India. Indian J. Surg. Oncol..

[42] Rubagumya F., Mitera G., Ka S., Manirakiza A., Decuir P., Msadabwe S.C., Ifè S.A., Nwachukwu E., Oti N.O., Borges H. (2020). Choosing Wisely Africa: Ten Low-Value or Harmful Practices That Should Be Avoided in Cancer Care. JCO Glob. Oncol..

[43] de Moraes F.Y., Marta G.N., Mitera G., Forte D.N., Pinheiro R.N., Vieira N.F., Gadia R., Caleffi M., Kauer P.C., de Camargo Barros L.H. (2022). Choosing Wisely for oncology in Brazil: 10 recommendations to deliver evidence-based cancer care. Nat. Med..

[44] Ting F.I., Uy C.D., Bebero K.G., Sacdalan D.B., Abarquez H.S., Nilo G., Ramos B., Sacdalan D.L., Uson A.J. (2022). Choosing Wisely Philippines: Ten low-value or harmful practices that should be avoided in cancer care. Ecancermedicalscience.

[45] Karim S., Benn R., Carlson L., Fouladbakhsh J., Greenlee H., Harris R., Henry N., Jolly S., Mayhew S., Spratke L. (2021). Integrative Oncology Education: An Emerging Competency for Oncology Providers. Curr. Oncol..

[46] Roumeliotis M., Morrison H., Conroy L., Becker N., Logie N., Grendarova P., Thind K., McNiven A., Hilts M., Quirk S. Competency-Based Medical Education in Radiation Therapy Treatment Planning. Pract. Radiat. Oncol..

[47] Fernandez C., Croke J., Alfieri J., Golden D.W. (2020). A guide to curriculum inquiry for brachytherapy simulation-based medical education. Brachytherapy.

[48] Naghi M., Salem M.R. (2021). A Modified Delphi Study for the Development of a Leadership Curriculum for Pediatric Oncology. Asian Pac. J. Cancer Prev..

[49] Song E.Y., Chuang J., Frakes J.M., Dilling T., Quinn J.F., Rosenberg S., Johnstone P., Harrison L., Hoffe S.E. (2021). Developing a Dedicated Leadership Curriculum for Radiation Oncology Residents. J. Cancer Educ..

[50] Aggarwal R., Swanwick T. (2015). Clinical leadership development in postgraduate medical education and training: Policy, strategy, and delivery in the UK National Health Service. J. Healthc. Leadersh..

[51] Turner S., Chan M.-K., McKimm J., Dickson G., Shaw T. (2018). Discipline-specific competency-based curricula for leadership learning in medical specialty training. Leadersh. Health Serv..

[52] Rosenblatt E., Ben Prajogi G., Barton M., Fidarova E., Eriksen J.G., Haffty B., Millar B.A., Bustam A., Zubizarreta E., Abdel-Wahab M. (2017). Need for Competency-Based Radiation Oncology Education in Developing Countries. Creat. Educ..

[53] Jalan D., Rubagumya F., Hopman W.M., Vanderpuye V., Lopes G., Seruga B., Booth C.M., Berry S., Hammad N. (2020). Training of oncologists: Results of a global survey. Ecancermedicalscience.

[54] Best L.R., Sengupta A., Murphy R.J., de Metz C., Trotter T., Loewen S.K., Ingledew P.-A., Sargeant J. (2019). Transition to practice in radiation oncology: Mind the gap. Radiother. Oncol..

[55] Khader J., Al-Mousa A., Al Khatib S., Wadi-Ramahi S. (2020). Successful Development of a Competency-Based Residency Training Program in Radiation Oncology: Our 15-Year Experience from within a Developing Country. J. Cancer Educ. Off. J. Am. Assoc. Cancer Educ..

[56] International A. Where We Are. https://www.acgme-i.org/about-us/where-we-are/.

[57] ICBME ICBME Collaborators—Roster. 2018 [Cited 2022 August 10]..

[58] World Health Organization Regional Office for Africa (2019). Core Competencies for the Eye Health Workforce in the WHO African Region.

[59] Republic of Kenya Ministry of Health (2019). Report of the Taskforce on Training of Medical Specialists.

[60] Rashid M.A. (2022). Hyperglobalist, sceptical, and transformationalist perspectives on globalization in medical education. Med. Teach..

[61] World Health Organization (2016). Global Strategy on Human Resources for Health: Workforce 2030.

[62] Pramesh C.S., Badwe R.A., Bhoo-Pathy N., Booth C.M., Chinnaswamy G., Dare A.J., de Andrade V.P., Hunter D.J., Gopal S., Gospodarowicz M. (2022). Priorities for cancer research in low- and middle-income countries: A global perspective. Nat. Med..

[63] Joint Royal Colleges of Physicians Training Board (2017). Specialty Training Curriculum for Medical Oncology.

[64] Royal College of Radiologists, Clinical Oncology Specialty Training Curriculum. 2021. [Cited 2022 August 10]. https://www.rcr.ac.uk/sites/default/files/clinical_oncology_curriculum_2021.pdf.

[65] Royal College of Physicians of Ireland Institute of Medicine, Higher Specialist Training in Medical Oncology, Farrington, K. Editor. Royal College of Physicians of Ireland, Ireland. 2022. [Cited 2022 August 10]. https://rcpi-live-cdn.s3.amazonaws.com/wp-content/uploads/2022/07/HST-Medical-Oncology-Curriculum-2022.pdf.

[66] The Accreditation Council for Graduate Medical Education, Hematology and Medical Oncology Milestones. 2019. The Royal Australasian College of Physicians, Physician Readiness for Expert Practice (PREP) Training Program: Medical Oncology Advanced Training Curriculum, The Royal Australian College of Physicians (RACP), Editor. First edition 2010 (Revised 2013). [Cited 2022 August 10]. https://www.racp.edu.au/docs/default-source/trainees/advanced-training/medical-oncology/medical-oncology-adult-medicine-advanced-training-curriculum.pdf?sfvrsn=75222c1a_8.

